# Depression, anger and coping strategies of students in polish medical faculties

**DOI:** 10.1192/j.eurpsy.2023.1150

**Published:** 2023-07-19

**Authors:** M. H. Wizner, A. Kawalec, N. Rodak, K. M. Wilczyński, M. Janas-Kozik

**Affiliations:** 1 Students’ scientific association at Department of Psychiatry and Psychoterapy of Developmental Age; 2 Department of Psychiatry and Psychoterapy of Developmental Age, Medical University of Silesia, Katowice; 3 Pediatric Centre of John Paul II, Sosnowiec, Poland

## Abstract

**Introduction:**

Stress related to high expectations towards students, a large amount of knowledge necessary to assimilate in a brief period of time, and peer pressure are an important factor in the deterioration of the mental state of medical students. As a consequence, it can lead to burnout and even the development of mental disorders such as depression. Mechanisms of coping with difficulties play an extremely important role in moderating this risk. For this reason, it was of the interest what strategies medical students adopt in the face of everyday stress and how it affects their well-being and functioning.

**Objectives:**

The objective was to determine how medical studies impact mental health of students and what coping strategies are used by them to mitigate the negative influence of stress associated with high expectations, peer pressure and overwork.

**Methods:**

A cross-sectional study was conducted among students of polish medical faculties using an online questionnaire. Risk of depression was assessed using validated BDI inventory, aggression using STAXI inventory and evaluation of coping strategies was conducted with Brief-COPE inventory.

**Results:**

Study was conducted among 329 participants. The majority of respondents were female (71.4%; n=235) and average age in the whole population equaled 22.46 years (95%CI: 22.1-23.01). There was no statistically significant difference in age between females and males. Average outcome in BDI equaled 13.84 (95%CI: 12.8-14.8) with higher levels among females (13.84 vs. 12 p<0.05). 165 (49.6%) students had a score above threshold for the increased risk of depression while 32 (9.63%) for severe symptoms of it. In case of aggression average outcome of STAXI equaled 24.89 (95%CI: 22.6-27.1). There was a statistically significant correlation between STAXI and BDI (r=0.3; p<0.05). In terms of the coping mechanisms in terms of coping strategies, a clear advantage of approach strategies was observed (65.36% of respondents). In the multiple regression analyses coping strategies did not influence neither STAXI nor BDI outcomes.

**Conclusions:**

What draws attention are the high level of depression among the surveyed students, where over 50% show results above the cut-off point for an increased risk of a depressive episode. The advantage of approach strategies is also interesting, especially in terms of planning strategies and positive reformulation. Interestingly coping strategies in the analysed population did not constitute a significant protective factor in relation to the severity of the depressive symptoms and agression. Meanwhile, the sense of satisfaction and contentment with the chosen direction was a very good protective factor in terms of the severity of depressive symptoms.

**Disclosure of Interest:**

None Declared

